# Decreased Serum EGF in First-episode and Chronic Schizophrenia Patients: Negative Correlation with Psychopathology

**DOI:** 10.1038/s41598-020-63544-0

**Published:** 2020-04-16

**Authors:** Xiaobin Zhang, Wenhuan Xiao, KuanYu Chen, Yaqin Zhao, Fei Ye, Xiaowei Tang, Xiangdong Du

**Affiliations:** 1 Institute of Mental Health, Suzhou Psychiatric Hospital, The Affiliated Guangji Hospital of Soochow University, Suzhou, Jiangsu 215137 P.R. China; 2 School of mental health, Jining medical University, Jining, 272000 P.R. China; 3 Department of Psychiatry, Affiliated WuTaiShan Hospital of Medical College of Yangzhou University, Yangzhou, 225003 P.R. China; 4 Department of Nursing, Huzhou University, Huzhou, Zhejiang, 313000 China; 5 Nanjing Brain Hospital, Nanjing Medical University, Nanjing, Jiangsu 210029 China

**Keywords:** Neuroscience, Human behaviour

## Abstract

Previous studies have demonstrated that neurotrophic factors may play a critical role in the severity of clinical symptoms in schizophrenia. However, it remains unknown whether serum levels of epidermal growth factor (EGF) in schizophrenia are similar to those observed in the case of other neurotrophic factors. Therefore, we compared serum EGF concentrations in first-episode drug-naive (FEP) patients and medicated chronic schizophrenic patients with healthy controls in order to explore whether EGF levels are related to psychopathological symptoms. We measured the serum levels of EGF in 78 first-episode medication-naive schizophrenia patients, 76 medicated chronic schizophrenic patients, and 75 healthy controls using the sandwich ELISA method. Disease severity were measured using the positive and negative syndrome scale (PANSS). Serum EGF levels showed a significant decrease in schizophrenia patients in comparison to healthy subjects. Serum EGF levels in FEP patients are indistinguishable from chronic cases. EGF levels were related to PANSS general symptom subscales in both FEP never-medicated and medicated patients. It is interesting that serum EGF levels were negatively correlated with the PANSS cognitive subscales, with the exception of the patients with chronic schizophrenia. Our preliminary results indicated that EGF may play a role in this illness and that it could be used as a potential biomarker of disease severity. Moreover, EGF may be associated with cognitive subscales of PANSS in FEP patients. Future studies should investigate the relationship between EGF and cognitive function as measured using standardized neuropsychological assessments to identify potential biomarkers related with cognition.

## Introduction

Epidermal growth factor (EGF), which was isolated from the submandibular gland of male mice by Stanley Cohen in 1962, is a very important neurotrophic factor, and is closely related to the proliferation, growth, differentiation, and regeneration of the central nervous system cell^1^. EGF exerts many biological functions by binding to its receptor EGFR and increasing Ca2+ concentration and the pH value in intracellular, which provides the necessary conditions for DNA synthesis and cell division^2,3^. Animal experiments showed that EGF could promote the growth of hippocampal neurons *in vitro* and enhance the activity of succinate dehydrogenase in neural stem cells^3–5^. In addition, EGF is a broad-spectrum neurotrophic factor which can ensure the survival of neurons, protect dopaminergic neurons from glutamate toxicity^6^, and repair neurons in the case of pathological conditions^2^, which is implicated in the repair process following brain injury.

There is evidence that EGF levels are associated with the pathogenesis of schizophrenia^7–12^. Genetic studies revealed a significant association between EGF gene polymorphism and premorbid and current cognitive functioning or the age of onset of schizophrenia^13–15^. In addition, peripheral transcription of NRG-ErbB pathway genes are upregulated in the case of treatment-resistant schizophrenia^16^. However, in a population-based sample in Japan, another study failed to support the hypothesis that EGF polymorphism is associated with schizophrenia^17^. A report by Futamura *et al*.^18^ provides the most convincing evidence to date that both the prefrontal cortex and striatum have lower EGF mRNA expression and higher EGFR expression in the prefrontal cortex in postmortem brain specimens from individuals suffering from schizophrenia. Moreover, recent research demonstrated that EGF could reduce the damage to hippocampal CAI neurons after transient cerebral ischemia^19^, which may be related to the inhibition of free radical-induced peroxisomal damage. These studies indicate disruption of EGF in the pathogenesis of schizophrenia.

Recently, investigators who examined the relationship between peripheral EGF levels and the psychopathology of patients with schizophrenia^8,18,20^ yielded conflicting results. A survey conducted by Futamura *et al*.^18^ revealed a significant decrease in serum EGF levels in the patient sample (i.e., 4 medication-naive and 45 medicated chronic schizophrenia patients) relative to healthy controls. These findings contrast with Hashimoto’s result that^21^ no differences were observed for serum levels of EGF in drug naïve, first-episode (n = 15) and chronically medicated patients (n = 25) with schizophrenia versus general population controls. Moreover, these studies were performed using a small sample size, as described above. Interestingly, the most recent research shows that plasma EGF levels were associated with cognitive decline in Parkinson’s disease^22–24^ and Alzheimer’s disease^22^. Until recently, there has been no reliable evidence to indicate that peripheral EGF is implicated in the cognitive functioning of schizophrenia patients.

Therefore, we aimed to compare serum EGF levels among a large cohort of chronic and acute schizophrenia patients in order to determine whether a correlation exists between EGF and psychopathological symptoms. Based on a growing body of literature examining the role of neurotrophic molecules in the pathophysiology of schizophrenia, we hypothesized that serum EGF levels were significantly diminished in patients with schizophrenia compared with healthy subjects. Moreover, we hypothesized that those with more serious psychiatric symptoms would show lower serum EGF levels, and a significant relationship between EGF and PANSS cognitive subscales was observed among patient groups.

## Experimental Procedures

### Participants, assessment, study procedures

154 patients aged 18 or above, who satisfied the criteria for schizophrenia, were recruited from an inpatient clinic at Yangzhou Wutaishan Hospital. All patients were Chinese. Each subject had been diagnosed and assessed independently by at least two of the authors according to the DSM-IV criteria based on structured clinical interview. 78 never-medicated first-episode psychotic (FEP) (male/female = 40/38) and 76 chronic schizophrenic patients treated with antipsychotics (male/female = 37/39) participated. Existing medications at baseline remained unchanged for at least six months before entry into the study, whereas initiation of other psychotropic medications was not permitted during the trial. A total of 57 patients were administered atypical antipsychotics alone, while 19 patients received a combination consisting in other typical agents. The information related to the participants’ medication use is as follows: aripiprazole (n = 8, 10.53%), amisulpride (n = 7, 9.21%), clozapine (n = 5, 6.58%), olanzapine (n = 9, 11.84%), risperidone (n = 11, 14.47%), quetiapine (n = 10, 13.16%), and ziprasidone (n = 7, 9.21%), clozapine-risperidone combination (n = 7, 9.21%), clozapine-aripiprazole combination (n = 6, 7.89%), clozapine-quetiapine combination (n = 6, 7.89%). The mean daily oral antipsychotic dose consisted in chlorpromazine equivalents (492.87 ± 203.89 mg/day) for chronic cases (Table 1).

**Table 1:** Demographics of participants.

	CS (n = 76)	FEP (n = 78)	HC (n = 75)	*F*/*t*/*χ*^2^	*P*
Age (years)	37.49 ± 11.14	26.03 ± 6.45	37.51 ± 12.3	32.269	**0.000**
Sex (Males, %)	37/39	40/38	42/33	0.831	0.660
Education (years)	9.67 ± 3.84	10.01 ± 3.18	12.51 ± 3.85	13.735	**0.001**
Smoking (yes,%)	27/49	28/50	33/42	1.466	0.481
Age of onset (years)	23.99 ± 8.46	24.29 ± 5.27		0.270	0.787
Duration of illness (years)	13.50 ± 9.84	1.73 ± 2.20		10.182	**0.000**
family history of mental illness(yes,%)	16(21.05)	17(21.79)		0.013	0.911
PANSS Total Score	78.88 ± 6.52	79.09 ± 5.04		0.221	0.825
Positive subscore	23.91 ± 5.82	24.24 ± 8.47		0.287	0.774
Negative subscore	24.07 ± 6.48	21.42 ± 8.03		−2.251	**0.026**
General subscore	30.91 ± 6.61	33.42 ± 3.59		2.924	**0.004**
Cognitive subscore	12.12 ± 2.82	11.37 ± 3.77		−1.393	0.166
**Atypical antipsychotics alone (n,%)**					
Aripiprazole	8(10.53)				
Amisulpride	7(9.21)				
Clozapine	5(6.58)				
Olanzapine	9(11.84)				
Risperidone	11(14.47)				
Quetiapine	10(13.16)				
Ziprasidone	7(9.21)				
**A combination of antipsychotics (n,%)**					
Clozapine-risperidone combination	7(9.21)				
Clozapine-aripiprazole combination	6(7.89)				
Clozapine-quetiapine combination	6(7.89)				
chlorpromazine equivalent (mg/day)	492.87 ± 203.89				

Abbreviations: FEP = never-medicated first-episode psychotic; CS = chronic schizophrenic patients; HC, healthy control; PANSS, Positive and Negative Syndrome Scale; Bold P-values indicate significant results.

A control group of 75 unmedicated healthy volunteers (male/female = 42/33) was included after interview with the SCID IV-NP (for non-patients). Any patient who had any personal or family history of any mental disorder or uncontrolled systemic disease were excluded. All subjects provided their written, informed consent to participate in the study, which was approved by Wutaishan Hospital Ethics Committee and Yangzhou University Medical Ethics Committee.

### Clinical assessment

On the day of blood sampling, three psychiatrists used the positive and negative syndrome scale (PANSS), with an intraclass correlation coefficient (ICC) of 0.80, as an outcome measure of patient’s psychopathology^25^. The five-factor PANSS model was used to assess the psychopathology of patients. This model includes positive, negative, excited, cognitive, and depressed factors. In addition, the cognitive factor of the model includes three items (P2, N5, G11) and is a valid measure of cognitive deficits in schizophrenia^26–28^.

### Serum EGF measurements

Blood samples were obtained prior to the meal. Samples were then immediately centrifuged at 3000 × g for 15 min, serum separated, aliquoted into small microcentrifuge tubes and stored at −70 °C until assayed. Sandwich enzyme-linked immunosorbent assay (ELISA) kits (R&D Systems, Minneapolis, MN, USA) were used to measure EGF in the sera of patients and controls. From this assay, the minimum detectable concentrations of EGF were 0.201 pg/ml, the intra-assay coefficient of variation (CV) was less than 5% and the inter-assay CV less was than 8%, respectively. The method of measuring serum EGF was the same as that previously described^9,21^. A technician who was blind to the clinical situation assayed all samples.

### Statistical analysis

The normality of data distribution was assessed using a histogram and Q-Q plots. As EGF was not normally distributed, we log-transformed EGF values. Differences between groups were analyzed using the Chi square test and t-test or variance analysis. We used Pearson’s correlation coefficient to calculate the correlation between groups. Moreover, linear regression was performed to examine the relationship between EGF and age, gender, duration of illness, age of onset, and the PANSS as well as its subscales. All statistical analyses were executed by IBM SPSS Statistics 23.0 (IBM Corp., Armonk, NY). Graphs were designed using Graphpad Prism 7.0 for Windows.

### Informed consent

Informed consent was obtained from all individual participants included in the study.

### Ethical approval

All procedures performed in studies involving human participants were in accordance with the ethical standards of the institutional and/or national research committee and with the 1964 Helsinki declaration and its later amendments or comparable ethical standards.

## Results

### Demographic data

The clinical and demographic characteristics of the three groups are presented in Table 1. Significant differences were found between these three groups with regard to age (P = 0.001) and education (P = 0.001). However, no significant differences were observed between patients and healthy controls in respect to gender and smoking (P > 0.05). Furthermore, PANSS total scores, as well as positive and cognitive subscores and family history of mental illness in the FEP group did not show any differences when compared with chronic medicated patients (P > 0.05). The t-test showed that PANSS negative scores were significantly higher and the general psychopathology subscores were significantly lower and length of illness was significantly longer in chronic patients than in the FEP group (P < 0.05). Afterwards, the variables that showed statistically significant differences between groups were added as co-variates in all subsequent analyses.

### Serum EGF levels

**Figure 1:**
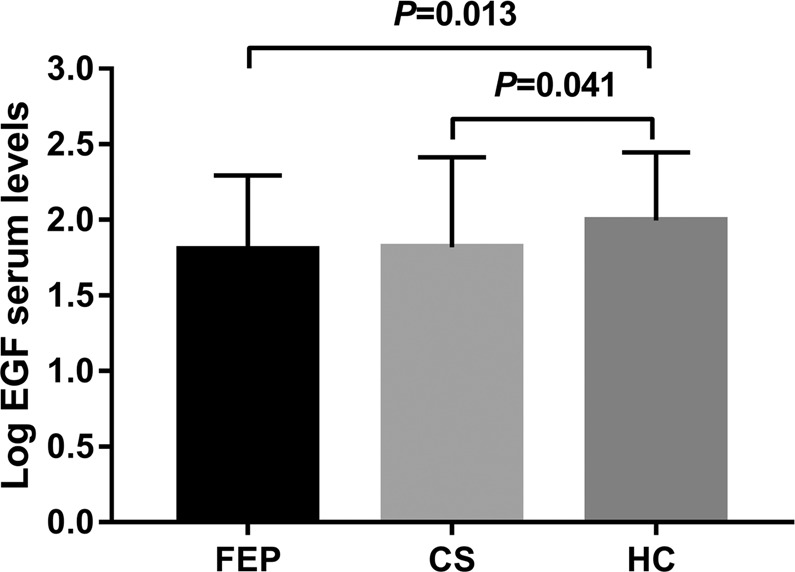
Log EGF serum levels in FEP and CS and HC. FEP = never-medicated first-episode psychotic; CS = chronic schizophrenic patients; HC, healthy control; EGF, endothelial growth factor.

Using an analysis of variance with the Bonferroni correction procedure, significantly lower serum Log EGF levels were found in FEP patients (1.80 ± 0.49) and medicated patients (1.82 ± 0.60) than in control subjects (1.99 ± 0.45), as depicted in Fig. 1. The results did not change after controlling for age (F = 2.862, df = 2, P = 0.059) or education (F = 2.474, df = 2, P = 0.087). When we compared the serum EGF levels of first-episode schizophrenia patients to those observed in chronic schizophrenia patients, no statistically significant difference was found (t = −0.162, df = 1, P = 0.872). Additionally, in respect to gender, there was no significant difference in EGF levels (t = 1.198, df = 1, P = 0.232).

### EGF and clinical and demographic characteristics

**Figure 2:**
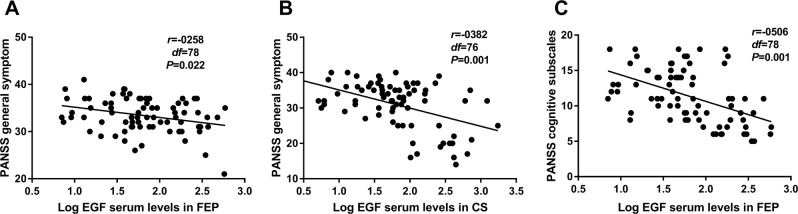
(**A**) Correlation between serum Log EGF levels and PANSS general symptom subscales in FEP (**B**) Correlation between serum Log EGF levels and PANSS general symptom subscales in CS. (**C**) Correlation between serum Log EGF levels and PANSS cognitive subscales in FEP. FEP = never-medicated first-episode psychotic; CS = chronic schizophrenic patients; HC, healthy control; PANSS, Positive and Negative Syndrome Scale; EGF, endothelial growth factor.

Pearson’s correlation coefficients between log-transformed serum EGF clinical and demographic characteristics were estimated for each group, as shown in Table 2. The observed EGF levels were undetermined by age, gender, education or smoking status in the patient population or controls (P > 0.05). As expected, a negative correlation was found between the PANSS general symptom subscales and the serum EGF levels in both never-medicated FEP (r = −0.258, P = 0.022) and medicated patients (r = −0.382, P = 0.001), as depicted in Fig. 2A,B. It is worth noting that a significant correlation was found between the PANSS total scores and serum EGF levels, though only in medicated patients (r = −0.421, P = 0.001). In addition, EGF was negatively related to PANSS cognitive subscales, though only in the FEP group (r = −0.506, P < 0.001) as depicted in Fig. 2C. In respect to serum EGF levels, no significant differences were found in terms of the different types of antipsychotics prescribed (r = −0.112, P = 0.338). No significant correlation was found while considering EGF levels and the type of antipsychotic, medication regimen or chlorpromazine equivalents used by the chronic patient group (P > 0.05). Moreover, EGF was not related with duration of illness and familial history of mental illness in both groups (P > 0.05).

**Table 2:** Correlations of demographic and clinical variables with serum log EGF levels.

	FEP (n = 78)			CS (n = 76)			HC (n = 75)		
	r	df	P	r	df	P	r	df	P
Age (years)	−0.070	78	0.545	−0.051	76	0.659	0.102	75	0.382
Sex (Males)	−0.128	78	0.266	−0.212	76	0.066	0.060	75	0.612
Education (years)	0.072	78	0.533	0.165	76	0.154	−0.119	75	0.308
Smoking (yes)	0.028	78	0.804	0.012	76	0.919	−0.124	75	0.289
Age of onset (years)	−0.094	78	0.413	0.023	76	0.844	—		
PANSS Total Score	−0.009	78	0.938	−0.421	76	**0.001**	—		
Positive subscore	−0.067	78	0.558	−0.048	76	0.681	—		
Negative subscore	0.181	78	0.112	0.090	76	0.438	—		
General subscore	−0.258	78	**0.022**	−0.382	76	**0.001**	—		
Cognitive subscore	−0.506	78	**0.000**	−0.091	76	0.435	—		

Abbreviations: FEP = never-medicated first-episode psychotic; CS = chronic schizophrenic patients; HC, healthy control; PANSS, Positive and Negative Syndrome Scale; Bold P-values indicate significant results.

To further assess the association between EGF and symptoms, multiple linear regression analyses were performed separately for each patient group. The findings of this stepwise analysis indicated that serum EGF levels were independently associated with the PANSS general psychopathology subscale in FEP patients (beta = −0.340, P = 0.021) and chronic patients (beta = −0.488, P = 0.001). Moreover, serum EGF levels in patients with first-episode schizophrenia were independently correlated with cognitive function (beta = −0.459, P = 0.001). However, the correlation between PANSS total scores and serum EGF levels in chronic patients was not statistically significant after controlling for clinical and demographic variables (beta = −0.271, P = 0.540), as shown in Table 3.

**Table 3:** Multivariate regression analysis showing an independent association between serum EGF levels and clinical symptoms*.

	FEP (n = 78)			CS (n =76 )		
	Beta	P	95%CI	Beta	P	95%CI
PANSS Total Score	0.099	0.414	−0.014–0.033	−0.271	0.540	−0.086–0.030
Positive subscore	−0.045	0.694	−0.016–0.011	−0.146	0.611	−0.066–0.039
Negative subscore	0.034	0.746	−0.011–0.015	−0.082	0.512	−0.015–0.030
General subscore	−0.340	0.021	−0.078–0.015	−0.488	0.001	−0.063–0.025
Cognitive subscore	−0.459	0.001	−0.088–0.031	−0.182	0.128	−0.088–0.011

^*^after controlling for clinical and demographic variables.

FEP = never-medicated frst-episode psychotic; CS = chronic schizophrenic patients; PANSS, Positive and Negative Syndrome Scale.

## Discussion

This is the first study to explore the correlations between serum EGF levels and clinical symptoms in medication-naive patients, chronic cases, and healthy controls. Blood levels of EGF were reduced in both medicated and drug-naive patients with schizophrenia compared to control subjects. Moreover, in our preliminary study, we found a close relationship between EGF and the PANSS general psychopathology and cognitive subscore in drug-naive patients with schizophrenia. Furthermore, we also found a significant negative association between EGF and PANSS general psychopathology in subjects treated with antipsychotic medication.

First, we found that serum EGF levels were markedly decreased in patients including first-episode and chronic patients. Our results are consistent with previous literature^18^ that suggested that both drug-naive and medicated chronic patients had a significantly lower mean EGF level than healthy subjects. However, these results contradict the findings of a Japanese study conducted in 2005^21^, which suggested that EGF levels were similar in the three groups. The discrepancy with our results can be reconciled by the different sample sources. Our findings support the theory that lower EGF levels may lead to schizophrenia, thus lending credence to the “neurotrophic factor hypothesis” which posits that schizophrenia is the behavioral outcome of aberration in the neurotrophic factor involved in neurodevelopmental processes.

Furthermore, we found that serum EGF levels were related to the PANSS general psychopathology subscore of schizophrenia patients. Such results are consistent with previous literature^21^ that suggests a close relationship between EGF concentrations and psychiatric symptoms as measured using BPRS for the entire patient group. In addition, Balotsev *et al*.^8^ showed that EGF serum levels are higher in patients with chronic schizophrenia than those of age-matched normal control subjects. Futamura *et al*.^18^ reported significantly decreased serum EGF concentrations in antipsychotic-free patients and in the antipsychotic-naive patients with schizophrenia. Recently, Haring *et al*.^20^ reported that serum levels of EGF were increased in FEP patients, but after for approximately 7 months antipsychotic medication treatment, EGF levels decrease and are related to the clinical improvement of FEP patients. It is well known that EGF protects dopaminergic neurons from glutamate toxicity in culture^6,16,29,30^. Serum EGF levels in patients were lower or higher than in the control group during all the stages of the illness, suggesting that dopaminergic neurons, in the case of patients, were obviously abnormal, and this abnormality is implicated in the mental symptoms and pathogenesis of schizophrenia.

The most interesting findings from this study highlight the fact that in drug-naive first episode schizophrenia patients, an association was found between EGF levels and PANSS cognitive subscale score, thus indicating that abnormal EGF levels may be a marker of cognitive deterioration. Nevertheless, the exact nature of this association remains unknown. In the brain, EGF family members serve as neurotrophic molecules to enhance stem cell proliferation and neuronal differentiation^31^, while influencing synaptic plasticity, including long-term hippocampal potentiation^2,32^. Moreover, recent experimental evidence suggests that EGF prevents APOE4 and amyloid-beta-induced cognitive and cerebrovascular deficits and promotes cognitive behavioral recovery in female mice^33^. Also, a disruption of ErbB signaling (EGF receptor) in adolescence increases striatal dopamine levels and affects learning as well as hedonic-like behavior in adult mice^34^. Specifically, lower EGF levels decrease hippocampal synaptic plasticity and impair cognitive function^19,31,35^. Interestingly, human studies have found that EGF levels were also associated with cognitive function in patients with Alzheimer’s^22^ and Parkinson’s disease^23^, further supporting the role of the EGF in cognitive impairment. Based on these findings, we speculation that EGF may be a useful biomarker for cognitive dysfunction. However, our study measured only a basic cognitive status, and did not employ any other methods to measure different types of cognitive functioning. Thus, the results offer only a partial insight into the relationship between EGF and PANSS cognitive subscales in schizophrenia. Furthermore, there is a need to carry out a cohort investigation the relationship between EGF and cognitive function assessed by a battery of cognitive tests covering aspects of memory, executive function and attention in patients with schizophrenia.

There were some limitations in this study. First, our study is limited by a relatively small sample size. However, this is only a preliminary study and larger studies are required to lend support to the findings of our research. Second, a cross-sectional survey, which was conducted to investigate the relationship between EGF levels and psychopathology, cannot establish a direct causal relationship. Third, since PANSS was designed specifically for psychopathology of schizophrenia. Although some items in the scale has been suggested to potentially represent “cognition” but they are not the cognition which commonly mentioned by schizophrenia research. Future studies should investigate the relationship between EGF and cognitive performance assessed by standardized neuropsychological assessments to help identify potential biomarkers for improved cognition.

In conclusion, our preliminary results demonstrate that in FEP and chronic schizophrenia, the serum EGF levels were markedly lower than those of control groups. Moreover, serum EGF is associated with psychopathology. These results indicate that EGF may play a role in this illness and that it could potentially be used as a biomarker of disease severity, despite the fact that the exact mechanisms are not yet fully understood.

## Data Availability

The datasets generated during and/or analyzed during the current study are available from the corresponding author on reasonable request.
